# Influence of Print Settings on the Critical Quality Attributes of Extrusion-Based 3D-Printed Caplets: A Quality-by-Design Approach

**DOI:** 10.3390/pharmaceutics13122068

**Published:** 2021-12-03

**Authors:** Silke Henry, Lotte De Wever, Valérie Vanhoorne, Thomas De Beer, Chris Vervaet

**Affiliations:** 1 Laboratory of Pharmaceutical Technology, Ghent University, 9000 Ghent, Belgium; Silke.Henry@UGent.be (S.H.); Lotte.DeWever@ugent.be (L.D.W.); Valerie.Vanhoorne@ugent.be (V.V.); 2 Laboratory of Pharmaceutical Process Analytical Technology, Ghent University, 9000 Ghent, Belgium; Thomas.DeBeer@ugent.be

**Keywords:** fused deposition modeling, 3D printing, personalised medicine, zolpidem, design of experiments, infill

## Abstract

Extrusion-based 3D-printing is an easy-to-use, cheap manufacturing technique that could be used to produce tailored precision medicines. The technique has an almost unlimited versatility since a multitude of print parameters can easily be adapted. Unfortunately, little is known of the effect of these print parameters on the critical quality attributes of the resulting printlets. In this study, practical guidelines and means to adapt certain parameters in order to achieve the desired outcome (e.g., acceptable visual quality and flexible dosing) are stipulated for medical 3D-printing using a design-of-experiments approach. The current study aims at elucidating the effect of five print parameters (infill, overlap, number of shells, layer height and layer pattern) on the mechanical properties, dimensions, weight, porosity and dissolution characteristics of a fixed-size caplet consisting of Eudragit EPO (69.3%), Polyox WSR N10 (29.7%) and zolpidem hemitartrate (1%). In terms of the mechanical properties, 3D-printed caplets possessed anisotropy where the vertical compression strength and Brinell hardness exceeded the diametral strength. In general, all 3D-printed caplets possessed acceptable mechanical strength except for a small region of the knowledge space. Dimensional analysis revealed small, statistical significant differences between different runs, although the clinical relevance of this variation is likely negligible. The weight or dose of a caplet can be varied mainly using the infill and overlap and, to a lesser extent, via the layer height and number of shells. The impact on porosity was complicated as this was influenced by many factors and their interactions. Infill was the only statistically relevant factor influencing the dissolution rate of the current formulation. This study unravels the importance of the print parameter overlap, which is a regularly neglected parameter. We also discovered that small dose variations while maintaining the same dissolution profile were possible via modifying the overlap or number of shells. However, large dose variations without affecting the dissolution behaviour could only be accomplished by size modifications of the printlet.

## 1. Introduction

The use of personalised dosage forms instead of mass-produced tablets is strongly encouraged due to its benefits for efficient treatment with minimal side effects. Mass-produced dosage forms are economically favourable but typically represent only the dose required to generate a therapeutic response in the majority of the population. Such an approach is detrimental in terms of patient safety for certain age groups (e.g., young children or the elderly), for drugs with a narrow therapeutic index or drugs with a complex dosing regimen. In these cases, a solution could be offered by on-site production of small batches of tailored medicines.

However, in order to practically implement this personalised dosing strategy, a flexible production technique is required. Additive manufacturing or 3D-printing could be a suitable technique to produce such dosage forms with varying shape, size or drug combinations [1,2].

Additive manufacturing is a generic term for seven distinct technologies that produce a 3D-printed object in a layer-by-layer manner using computer-aided design (CAD) [2]:

(i) Vat polymerization or stereolithography employs a high energy light to polymerize and solidify a vat of liquid resin [3]. For example, osteochondral scaffolds or six-layer polypills have been prepared utilizing this technique [4,5].

(ii) In directed energy deposition, metal powder is molten with a high-power laser beam [6].

(iii) In binder jet printing (BJ), a binder liquid is sprayed onto a powder bed, which consequently agglomerates. Spiritam, the first FDA-approved 3D-printed tablet, was produced using this technique [7]. BJ can also be employed to print and adapt biomaterials to control cell growth and adhesion [8].

(iv) Sheet lamination is another technique where multiple sheets of, e.g., plastic, paper or metal foil are adhered to each other using adhesives [1]. This technique has thus far only been employed to construct medical models [9].

(v) Material jet printing utilizes a polymeric solution, which is dropped onto a build platform and subsequently solidifies through solvent evaporation or UV light drying [2,10]. Both drug-loaded systems and biodegradable scaffolds have been produced [6].

(vi) Powder bed fusion or selective laser sintering (SLS) employs a laser beam focussing on a powder bed and hence creating a sintered layer [2]. Due to its excellent resolution, SLS has been investigated extensively within the medical field to produce printlets with a variety of release profiles, implants or multi-reservoir systems [11].

(vii) Material extrusion includes both semi-solid extrusion and molten polymer extrusion (i.e., fused deposition modelling, FDM). Semi-solid extrusion utilizes disposable syringes at low printing temperature that allow sequential deposition of a gel or paste structure. Afterwards, solidification is achieved by cooldown, solvent evaporation or photopolymerisation [12].

FDM 3D-printing, on the other hand, employs a polymeric filament as a starting material, which is fed to a heated nozzle by means of two rollers [13]. Both techniques have been investigated in the medical field to produce scaffolds, implants and drug delivery systems [14,15,16]. While each of the discussed 3D-printing techniques has its unique advantages and disadvantages, FDM is generally regarded as the easiest and cheapest desktop printing technique favouring on-site production in a local point of care [15,17].

Unfortunately, the mechanism behind the FDM printing process places some constraints on the material properties of printable filaments. The roller gears within the print head pinch the filament and transport it towards the heated nozzle. Brittle polymers might be unable to withstand the pressure and break between these roller gears, while flexible polymers might be too elastic to overcome the pressure within the print head [18,19].

Alternatively, pellet or powder printing could avoid this limiting feeding step, which would enable printing with a wider range of materials [20,21]. After the filament has successfully passed the driver gears, both print speed and temperature are critical process settings to enable printing, as these settings are directly correlated to the pressure drop over the nozzle [13,18]. It has been noted that a change of these settings impacted, e.g., the resulting weight or porosity of the printlet [22,23].

In general, when a filament possesses the ideal mechanical properties to enable feeding, the process settings should be optimised in order to print a qualitative, reproducible dosage form. Since FDM 3D-printing is a CAD-based manufacturing technique, a multitude of slicer parameters can be varied even for a fixed-size structure. Among these slicer parameters are the infill, number of shells, layer height, overlap and layer pattern.

The infill (%) represents the number of lines within the tablet and can be varied from 0 to 100%. A tablet with 100% infill is completely filled and possesses a theoretical porosity of 0%. Such a high infill was found to be detrimental for the quality of the resulting caplet, especially on the rounded edges [24]. Infill was previously varied to create dosage forms with a range of drug release profiles [25,26]. Moreover, a very low infill (<20%) was employed to develop floating dosage forms [27]. The number of shells or perimeters are the number of outlines, which form the tablet wall. Mostly, drug delivery systems are produced with one or two shells [28,29,30] or occasionally more [31].

The layer height represents the thickness of each layer, which affects both the vertical resolution and printing time. The height itself is limited by the nozzle diameter [13,32]. In order to find a compromise between print speed and resolution, some dosage forms are produced with a layer height of 50% nozzle size [33,34], while others are produced at only 25% nozzle size to maximize the resolution of the printlet [29,30,35].

The overlap (%) represents the degree of overlap between the infill and shell but, thus far, this parameter has received little attention in the literature. Lastly, different fill patterns can be chosen to fill the printed structure, e.g., rectilinear, line, honeycomb or spiral [33]. Choosing a complex infill structure is often detrimental in terms of production time. The choice and combination of these listed settings is often made empirically: settings might be either fixed to compare different formulations [34,35,36], or settings might be varied in order to produce the most qualitative tablets [29]. While FDM 3D-printing is praised for this endless flexibility, the effect of its slicer settings on the critical quality attributes of the resulting dosage form is largely unknown.

In this research, the effect of the slicer settings on the critical quality attributes (CQA) of a 3D-printed caplet is investigated. As such, when the end-user adapts certain slicer parameters in order to modify the dose, outlook or dissolution behaviour of the caplet, the effect on other CQAs can be predicted instead of using a trial-and-error approach. The general CQAs of 3D-printed dosage forms are weight, dimensional accuracy, hardness and drug release profile [37].

The selection of these CQAs was made based on the general monograph for tablets in the European Pharmacopoeia (Ph.Eur.) 10.7. In this study, an oblong tablet (caplet) was chosen as investigated dosage form since tablets are the most accepted route of administration, and, in general, oblong and oval tablets are slightly easier to swallow than round tablets [38].

## 2. Materials and Methods

### 2.1. Materials

Zolpidem hemitartrate (ZHT) (Aarti Drugs, Mombasa, India) was used as the active pharmaceutical ingredient (API) and was blended with Eudragit EPO^®^ and Polyox WSR N10^®^ (1:69.3:29.7, w:w) to generate the feedstock material used for 3D-printing. The melting and degradation of ZHT are initiated at 188 ${}^{\circ }C$ and 190 ${}^{\circ }C$, respectively. Eudragit EPO^®^ was kindly gifted by Evonik (Hanau, Germany) and is an amorphous methacrylate-copolymer with an approximate molecular weight of 47,000 g/mol and a glass transition temperature of 52 ${}^{\circ }C$.

Polyox WSR N10^®^ was kindly gifted by Dupont (Hamm, Germany) and is a semi-crystalline, non-ionic polyethylene oxide with an approximate molecular weight of 100,000 g/mol, and a glass-transition and melting temperature of −67 ${}^{\circ }C$ and 65 ${}^{\circ }C$, respectively. Both polymers are approved for pharmaceutical use. Scotch blue painter’s tape 50 mm was supplied by 3M (Bracknell, UK).

### 2.2. Filament Preparation by Hot Melt Extrusion

Polymer systems consisting of Eudragit EPO (EPO) and Polyox WSR N10 (PEO) were blended with ZHT and extruded to generate feedstock filament for FDM 3D-printing. EPO was chosen as main polymer component as it displayed favourable thermal and rheological properties for 3D printing in combination with excellent stability due to its low hygroscopicity as discussed in a previous paper [24]. PEO was added to the formulation to enhance the mechanical properties of the filament and to enable feeding [24].

Extrusion was performed using a co-rotating, fully intermeshing twin-screw extruder (Prism Eurolab 16, Thermo Fisher, Dreieich, Germany) equipped with co-rotating twin screws and a custom-made heated die of 1.70 mm diameter. A QT-20 feeder (Coperion, Niel, Belgium) was used at a feed rate of 0.3 kg/h. The screw speed was kept constant at 40 rpm and temperature was set at 140 ${}^{\circ }C$ and 120 ${}^{\circ }C$ for the barrel and die, respectively.

These settings were previously optimized and generated a stable extrusion process with a torque of approximately 35% of the maximal achievable torque [24]. A screw configuration consisting of transporting elements, two kneading blocks and a discharge element were used [39]. Resulting filaments were collected on a self-winding roller with adapted speed to obtain filaments with a diameter of 1.75 ± 0.05 mm as measured with a digital caliper. Filaments with a diameter out of this range were discarded [24].

### 2.3. Extrusion-Based 3D-Printing of Caplets

Caplets with 11 mm length, 4.4 mm height and 5.5 mm width were designed as a .stl file using OnShape and converted into G-codes using the Slic3r Prusa Edition software (Prusa Research, Prague, Czech Republic). All caplets were printed using a Prusa i3MK3S (Prusa, Czech Republic) with one top/two bottom layers and an extrusion multiplier of 1. The first layer of the caplet was printed with a speed of 10 mm/s and other layers at a speed of 50 mm/s. A fan, blowing on the printed object, was disabled during the first layer and enabled at 100% of its maximum speed during the consecutive layers. All caplets were printed with a nozzle temperature of 160 ${}^{\circ }C$, bed temperature of 55 ${}^{\circ }C$ and nozzle size of ∅0.40 mm.

### 2.4. Design of Experiments

The effect of five print settings (factors) on caplet properties was evaluated using a fractional factorial design (resolution V+) with 20 experiments using four replicates. Investigated factors were (i) the amount of infill (%), (ii) overlap (%), (iii) number of shells, (iv) layer height and (v) infill pattern (line or honey comb) as can be seen in Table 1. This subset of parameters included in the experimental design was chosen based on previous work (e.g., the importance of a parameter to generate a qualitative tablet) and based on a literature screening (e.g., parameters that were most often selected as variables in research articles).

Regarding the infill, the line and honeycomb patterns were selected from a list of 13 available patterns as these were distinctively different from each other in comparison with, e.g., rectilinear, line or grid pattern. The experimental ranges were determined based on preliminary trials in such a way that parameter settings generating defective caplets were avoided (e.g., minimal infill of 20%). The individual experiments were performed in a randomized order. Responses were regressed against the factors with multiple linear regression (MLR) using MoDDE 12.1 (Umetrics, Umeau, Sweden). Factors were scaled and centred, and 95% confidence intervals were calculated.

### 2.5. Caplet Characterization

#### 2.5.1. Mechanical Properties

**Figure 1:**
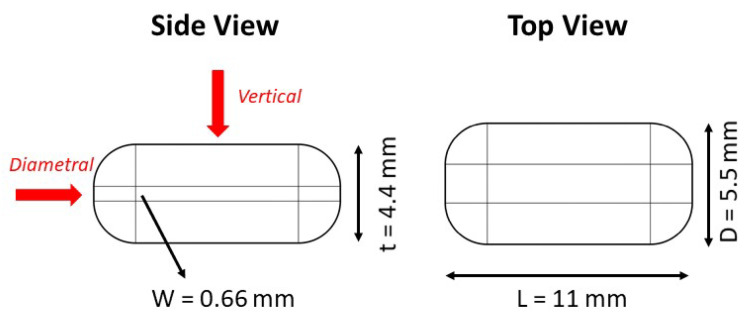
A graphical representation with dimensions of the fixed-size caplet used in this study. The two directions for the mechanical tests are displayed with red arrows.

The mechanical properties of the resulting caplets are of vital importance to withstand stress during manufacturing and during manipulation by the end user, as stipulated by USP monograph 1217. FDM 3D-printed products are highly anisotropic in comparison with traditional compacts. As a result, the mechanical tests as developed by the pharmacopeia (e.g., diametral) might not suffice for the characterization of 3D-printed caplets. As a result, the mechanical strength was assessed in the diametral and vertical direction as can be seen in Figure 1. In addition, the vertical Brinell hardness and elasticity were also assessed.

##### Diametral Compression

The diametral breaking force (N) of the caplets (n = 10) was determined using a hardness tester (Pharmatron SmartTest 50, Basel, Switzerland) in compliance with USP monograph 1217 and Ph.Eur. monograph 2.9.8 using flat-surfaced jaws crushing the caplet. The tensile strength of the caplet was calculated using the equation as described by Pitt and Heasley, which is a modified form to calculate the tensile strength of caplets based on the method described for tablets in USP monograph 1217 [40]:

(1) $$\sigma_{t}=\frac{2}{3}\frac{10P}{\pi D^{2}\left(2.84\frac{t}{D}-0.126\frac{t}{W}+3.15\frac{W}{D}+0.01\right)}$$

where $\sigma_{t}$ represents the tensile strength (MPa), *P* the breaking force (N), *D* is the width (mm), *t* is the thickness (mm) and *W* is the flat part (mm) of the convex caplet. These dimensions are 5.50, 4.40 and 0.66 mm, respectively, for the current caplet design as can be seen in Figure 1.

##### Vertical Compression

Caplets (n = 10) were subjected to a vertical compression test using a TA.HD PlusC Texture analyser equipped with a heavy duty platform and steel ball probe P/5S (Stable Micro Systems, Godalming, UK). Settings were optimised during preliminary screening. A ball probe moved into the specimen at a speed of 0.02 mm/s, and data logging was initiated after reaching a trigger force of 50 g. The force and area under the curve of the first peak were calculated using Matlab2018b.

##### Brinell Hardness

The Brinell hardness test was performed on the caplets (n = 10) using a TA.HD PlusC Texture analyser equipped with a heavy duty platform and steel ball probe P/5S (Stable Micro Systems, UK). Caplets were fixed on the platform using double-sided tape to prevent lifting from the platform when the load was removed. A force of 3 kg was held on the caplet for 80 s. The Brinell hardness was calculated as shown:

(2) $$BHN=\frac{F}{\pi Dh}$$

where *BHN* represents the Brinell hardness (kg/mm${}^{2}$), F is the applied force (kg), *D* is the ball probe diameter (5 mm) and *h* is the unloaded indentation depth (mm). Moreover, the elastic recovery (ER, %) was calculated by dividing the area under the unloading plot by the loading plot of the force (N) versus distance (mm) profile.

#### 2.5.2. Dimensional Analysis

Masses and dimensions (width, length, diameter) of the 3D-printed caplets (n = 10) were recorded using an automated tablet tester (SmartTest 50, Sotax, Basel, Switzerland).

#### 2.5.3. Porosity Analysis

The porosity of the caplets (n = 3) was calculated using the following equation:

(3) $$Porosity\left(\%\right)=1-\frac{V_{app}}{V_{env}}\times 100$$

where $V_{app}$ represents the apparent volume as determined by helium pycnometry (AccuPyc 1330, Micrometrics, Norcross, GA, USA) at an equilibration rate of 2.5738 kPa/min with the number of purges set to 10. $V_{env}$ represents the envelope volume, which was calculated for each run separately based on the dimensions measured with the tablet tester. For this calculation, we assumed that any dimension change occurred homogeneously throughout the caplet in each direction.

#### 2.5.4. Dissolution Behaviour

In vitro release experiments (n = 3) were based on the USP guidelines for zolpidem hemitartrate immediate release tablets. The content of tablets used for dissolution study is displayed in Table S1. A PTWS 120D dissolution system (Pharma test, Hainburg, Germany) equipped with sinker baskets and paddles set at a rotational speed of 50 rpm was used. The dissolution medium was 900 mL of sonicated 0.01 M HCl solution (pH 2) of which 5 mL samples were withdrawn at each time point and analysed spectrophotometrically (UV 1650PC, Shimadzu, Brussel, Belgium) at 295 nm. In vitro dissolution curves were fitted to a Weibull model:

(4) $$log\left[-ln\left(1-\frac{Q_{t}}{Q_{\infty }}\right)\right]=\beta log\left(t\right)-log\left(\alpha \right)$$

where *Q*${}_{t}$ is the amount of zolpidem dissolved at time *t*, *Q*${}_{\infty }$ is the amount of zolpidem dissolved at infinite time, α is the scale parameter and β is the shape parameter. The time interval necessary to dissolve 63.2% of the API present in the caplet is defined as the *T*${}_{d}$:

(5) $$T_{d}=\sqrt[{\beta }]{\alpha }$$

A dissolution test was preferred over a disintegration test due to its superior informative character and the solubility of the polymers. The Weibull model was chosen to fit the data since it is a widely applicable model, allowing to successfully fit a wide range of dissolution profiles. Moreover, the *T*${}_{d}$ parameter enables easy comparison between different dissolution profiles in current and future research experiments.

## 3. Results and Discussion

**Figure 2:**
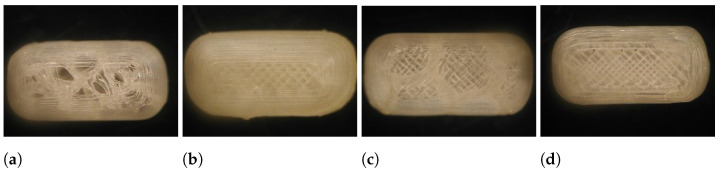
Different print settings resulted in visually different caplets. Caplets of run 1 (**a**), run 4 (**b**), run 5 (**c**) and run 9 (**d**) are shown.

All runs of the experimental design were successfully executed yielding caplets with distinctively different outlooks as can be seen in Figure 2. Raw data of the characterization tests can be found in Table S3. Some difficulties arose when caplets were printed with all factors set at their highest level as these caplets were not sticking properly to the heated bed.

### 3.1. Mechanical Properties of the Resulting Caplets

In extrusion-based 3D-printing, high density, tightly structured and smoothly surfaced products are generated since the printlets consist of solidified polymer strands. As a result, 3D-printed tablets usually possess high hardness when compared to directly compressed tablets with their breaking force often exceeding the upper limit of hardness testers [29,41]. In addition, the friability of the resulting tablets was often found to be (almost) zero [42,43,44]. Tablets with low hardness might, however, be produced when brittle filaments with low toughness are used as a starting material [29,45].

In general, the influence of the slicer settings on the tablet’s mechanical properties has been poorly investigated, and a deeper insight in this quality attribute would elucidate which regions of the knowledge space are accessible to generate tablets with sufficient mechanical strength [46]. It should be kept in mind that, unlike conventional directly compressed tablets, 3D-printed caplets are anisotropic, which means that the mechanical properties are different when measured along another axis [37,47].

Therefore, the tensile strength will be measured using localized vertical compression in addition to the standard diametral compression test. In order to maximally differentiate between caplets produced with different print settings, the hardness and elasticity were also measured using the Brinell test.

#### 3.1.1. Diametral Compression

During the standard diametral compression test, we noticed that tablets sometimes split upon compression or displayed deviation from a regular fracture pattern due to plastic deformation. This behaviour resulted in a higher relative standard deviation (RSD) for certain runs (e.g., RSD of 47.3% for run 16 or 47.9% for run 18). It is hypothesised that, while the diametral compression test is the preferred method for conventional compressed tablets, it is not the ideal test for products with higher elasticity.

It was shown previously that the test is not applicable to all tablets, and similar problems (e.g., no radial cracking and plastic deformation) arose when testing orally disintegrating tablets [48]. Another work reported orientation dependency of the diametral hardness when a circular tablet was used due to the inhomogeneous infill when the line pattern is chosen [37]. Such phenomena did not occur in the current research since oblong caplets were used, which could all be compressed in the same diametral direction.

**Figure 3:**
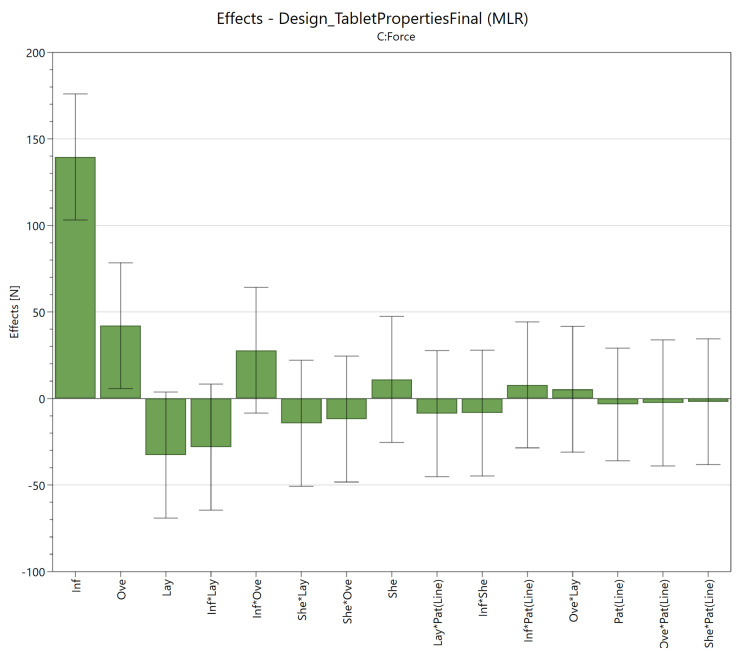
Effect plot of the diametral compression force including 95% confidence intervals for infill (Inf), overlap (Ove), number of shells (She), layer height (Lay) and infill pattern (Pat) with their interactions (*) displayed as factors.

The impact of the individual factors and their interactions on the responses are displayed in an effect plot as can be seen in Figure 3. This plot displays the change in response when the lowest level of a given factor (−1) is replaced with its highest level (+1), while all other factors are kept at their center point (0). This change corresponds to twice the MLR coefficient [49]. The significant factors (95% confidence interval) in the effect plot were the infill (139.5 N) and overlap (42.1 N).

A higher infill results in stronger tablets by augmenting the density, an observation consistent with literature findings [25,37,43]. Surprisingly, the overlap was also an important factor, possibly because it increases the level of bonding between the infill and the surrounding shells. Shell thickness did not seem to significantly influence the diametral hardness, which seems to contradict earlier research [37]. An explanation could be that Zhang et al. used circular tablets instead of the smaller caplets in the current study.

#### 3.1.2. Vertical Compression

A vertical compression test was developed due to the anisotropy of the 3D-printed caplets and the occurrence of flexible deformation during a conventional diametral compression test. When the caplet is compressed vertically using a ball probe, the initial peak force is followed by a series of minor fractures. While the exact fracture profile is irregular, the first peak force value is reproducible. The area under the curve (AUC) of this first peak is appointed as the toughness of the caplet, since it represents the ability of the caplet to absorb energy before its fracture. Both the peak force (N) and AUC (mJ) were investigated as responses, with the relative standard deviation being considerably lower for the peak force value (mean of 14.9%) than toughness values (mean of 27.8%).

The significant factors in the effect plot for both the peak force value and toughness were the infill (77.6 N or 41.5 mJ, respectively) and overlap (25.4 N or 19.5 mJ, respectively) as can be seen in Figure S1. These are the same factors as discovered during the diametral compression test.

#### 3.1.3. Brinell Hardness

**Figure 4:**
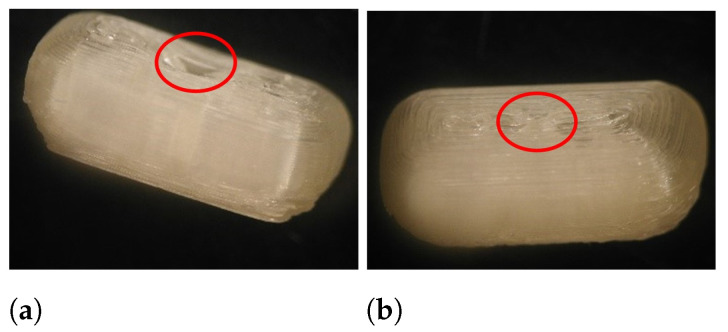
The load imposed on the caplet’s surface left an indentation of which the depth depends on the hardness of the product. A caplet of run 1 (**a**) and run 8 (**b**) is shown.

During the Brinell hardness test, a load was imposed on the caplet’s surface, which left an indentation as can be seen in Figure 4. Based on the depth of this indentation, the Brinell hardness was calculated. The elastic recovery (%) was calculated as the ratio between the area under the curve (AUC) of the unloading and loading plot.

Concerning the Brinell hardness, the significant factors were the infill (0.76 kg/mm${}^{2}$), layer height (0.11 kg/mm${}^{2}$) and overlap (0.10 kg/mm${}^{2}$). Additionally, the significant interactions were the layer height*infill pattern (0.25 kg/mm${}^{2}$), infill*overlap (−0.14 kg/mm${}^{2}$), overlap*layer height (0.13 kg/mm${}^{2}$) and overlap*pattern (0.11 kg/mm${}^{2}$). The effect plot and interaction plots can be found in the Supplementary Materials (Figures S1 and S2). The significant main effects, infill and overlap, can be explained similarly to the diametral and vertical compression test.

The layer height exerted only a small effect on the measured hardness. Most likely, a thicker layer cools down more slowly leaving more time for polymer sintering and interlayer bond formation [13]. For the layer height*infill pattern, the Brinell hardness was higher with the line pattern at increasing layer height, while the honey comb pattern had the opposite effect. This results from a different distribution of the infill within the caplet, dependent on the chosen pattern.

The line pattern was deposited in a more random manner, while each strand of the infill is put directly on top of each other in the honeycomb pattern. The overlap*infill pattern interaction was the result from an increase of the Brinell hardness with increasing overlap when the line pattern was used. When the honey comb pattern was used, only a negligible effect of the overlap existed. The honeycomb pattern acts as an extra shell within the caplet, thereby diminishing the effect of the overlap. Lastly, the effect of the overlap on the Brinell hardness was more pronounced at low infill.

**Figure 5:**
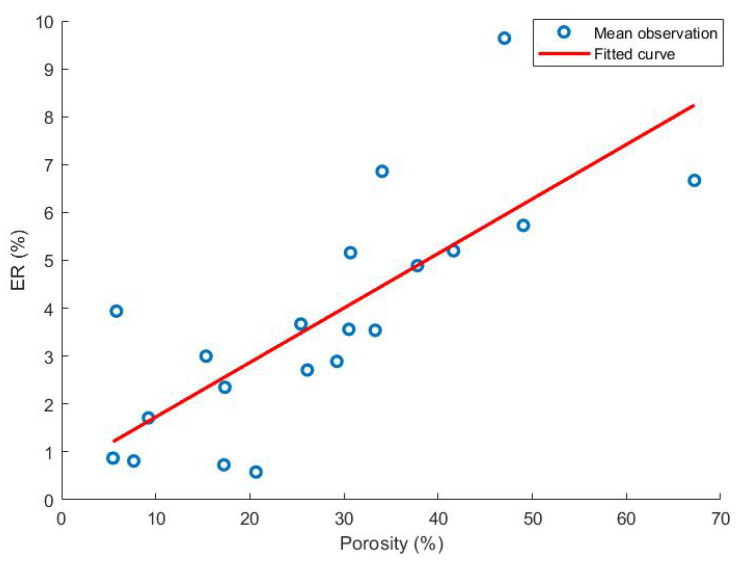
The correlation between the elastic recovery (%) and the porosity (%) as determined with helium pycnometry. Reported observations are the mean values of 10 (ER) or 3 (He pycnometer) repetitions for each run. First order linear fitting was performed, and an R${}^{2}$ of 0.61 was obtained.

The significant main effects of the elastic recovery data were infill (−4.03%) and layer height (−1.67%). From these effects, we deduced that caplets made out of thin strands with a low infill have the largest elastic recovery values. Based on this result, it is hypothesised that the elastic recovery is correlated with the caplet porosity. As can be seen in Figure 5, a linear correlation between both responses might be noticed.

The porosity itself is discussed later on, but the link between the mechanical properties and porosity was also described previously by Zhou et al., where a linear relationship was discovered between the bonding strength of dog bone shaped samples and the porosity calculated from the density of these samples [50].

In conclusion, the Brinell test captures the hardness and elasticity of the caplet and seems more sensitive to detect variations in print conditions by unravelling multiple interactions. Both hardness and elasticity seem to rely on multiple parameters and will, therefore, generate more complex models.

#### 3.1.4. Tensile Strength

In general, a tablet should possess a tensile strength greater than 1.7 MPa in order to withstand stresses induced by commercial production, transportation and handling [40,51]. Often, a strength of 2 MPa or even higher is pursued to ensure robustness of the end-product [51,52]. It has been anticipated, however, that tensile strengths of 1 MPa might also suffice for small batches that are not subjected to large mechanical stresses [51].

Supposedly, this lower tensile strength will also suffice for 3D-printed tablets as they are not subjected to f.e. industrial coating or packaging processes and could have a shorter chain between the production site and the patient. Unfortunately, no guidelines or regulations stipulating the minimal tensile strength for 3D-printed dosage forms exist yet. Therefore, in this study, a limit of 1.7 MPa tensile strength was chosen.

**Figure 6:**
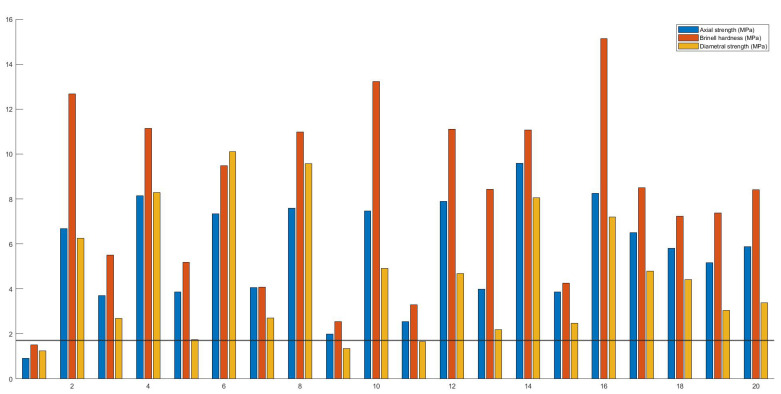
A comparison of the tensile strengths of FDM 3D-printed caplets as determined by diametral and vertical compression, and the Brinell hardness. The horizontal line at 1.7 MPa represents the minimal tensile strength.

Each run with their respective calculated vertical and diametral strength, and Brinell hardness are presented in Figure 6. Based on the vertical strength and Brinell hardness test, only caplets of run 1 were deemed too fragile (<1.7 MPa). This is in accordance with previous studies where 3D-printed dosage forms possessed excellent mechanical properties or even exceeded the maximal capacity of hardness testers [53]. When the results of the diametral compression were examined, additional failures for run 9 and 11 were observed. It is not unlikely that a 3D-printed tablet is more sensitive to failure under diametral than vertical load since the product is produced in a layer-by-layer manner.

Although the main objective of the study was to screen and investigate the most influential factors on each response, models were constructed for the Brinell hardness, diametral and vertical strength to display the usefulness of a quality by-design-(QbD) approach for caplet optimization. However, it must be noted that a more rigorous dataset with factors at multiple levels (e.g., 5 instead of 3) should be analysed in order to develop a fully optimized, qualitative model for prediction purposes.

The models were constructed by excluding terms with the smallest, insignificant effect for each response in a step-wise manner as long as the Q${}^{2}$ did not drop more than 0.1 units when compared to the maximal achievable Q${}^{2}$. The R${}^{2}$ of a model shows the model fit, the Q${}^{2}$ shows an estimate of future predictions, and the reproducibility shows the variation of the replicates compared to the variability over all the runs.

They all should be larger than 0.5 for a good model. Model validity could reveal model problems, like outliers or a transformation issue, when its value is below 0.25 [49]. For the diametral strength, the resulting model contained the following main effects: infill, overlap, layer height and the following interactions: infill*overlap and infill*layer height. The resulting model for the vertical strength contained the following factors: infill, shells, overlap (main effects) and shells*overlap (interactions).

**Figure 7:**
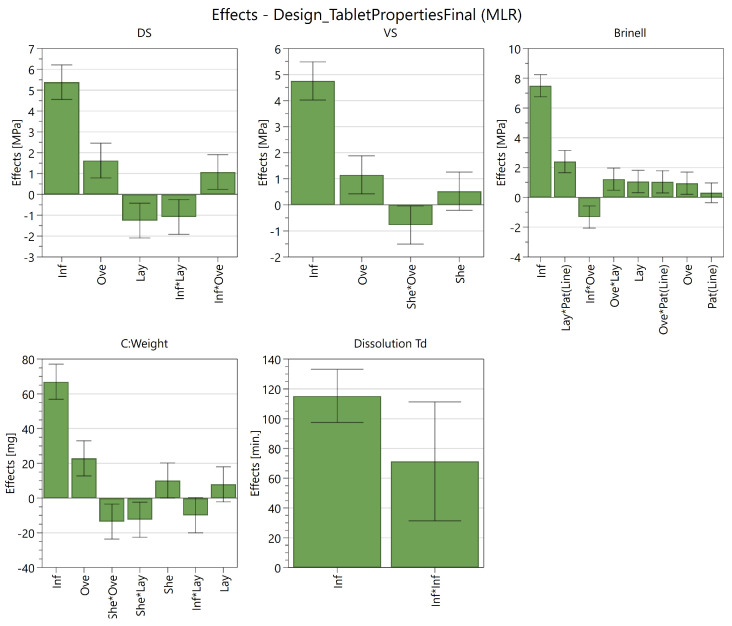
Optimized effect plots of the Brinell hardness, diametral (DS) and vertical strength (VS), weight and dissolution properties (T${}_{d}$) of the FDM 3D-printed caplets.

For the Brinell hardness, the resulting model contained the following main effects: infill, overlap, layer height, infill pattern and the following interactions: infill*overlap, overlap*layer height, overlap*infill pattern and layer height*infill pattern. A summary of the retained effects for these models can be found in Figure 7. A summary of fit for these models can be found in Table 2.

**Figure 8:**
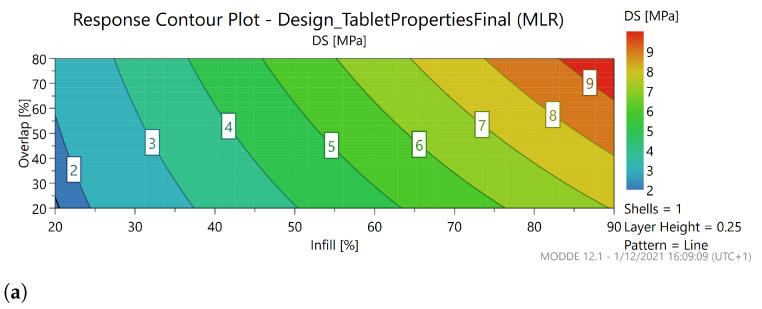

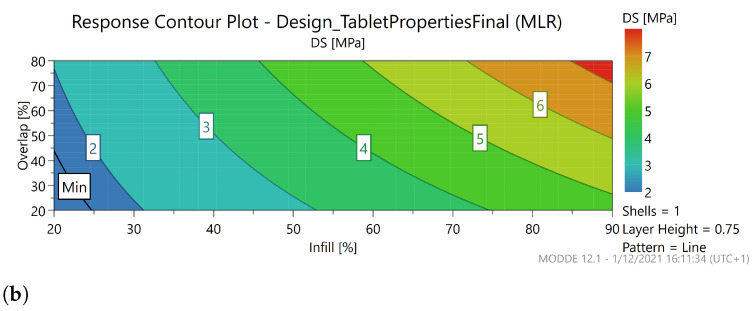
Contour plot of diametral strength (DS) as a function of the overlap (%) and the infill (%). The factor pattern is fixed at the line pattern, and the number of shells is 1. The layer height is either 0.1 (**a**) or 0.3 (**b**). At a layer height of 0.3 mm, a small region in the knowledge space is inaccessible (black line) since it generates caplets with defective mechanical properties (DS < 1.7 MPa).

A contour plot was constructed for the diametral strength (DS) as can be seen in Figure 8, since this is the limiting factor in terms of mechanical strength as can be seen in Figure 6. The pattern (line) and number of shells (1) were fixed, these do not influence the diametral strength. At high layer height (0.3), a region exists where DS is <1.7 MPa. It can be deducted from the contour plot that lower values of the infill should be avoided when combined with a low overlap at high layer height. For lower layer heights, almost no limitation exists, as all factor settings produced caplets with acceptable tensile strength. In conclusion, a very small region within the knowledge space is inaccessible, but most settings can be freely adapted to create caplets with acceptable mechanical properties.

### 3.2. Dimensional Analysis

All caplets were printed with the same in silico model and, hence, possessed the same theoretical dimensions of 11 mm length, 4.4 mm height and 5.5 mm width. In general, the actual dimensions of the 3D printed object might deviate from its theoretical dimensions due to polymer relaxation, which is a material-dependent property. For example, a tablet printed with HPC-L instead of HPC-SL based on the same in silico model gave a diameter increase from 9.8 to 10.4 mm [34]. In another study, smaller dimensions compared to the theoretical ones were reported for a variety of materials [37,54]. The influence of process settings on these dimension is less known and, therefore, investigated in-depth in the following section. All significant effects for the factors included in the dimensional analysis are displayed in Table S2.

#### 3.2.1. Diameter

For each run, the diameter was lower than 11 mm and varied between 10.62 and 10.93 over all runs. As such, the differences were small between different runs (maximally 0.31 mm), and the relevance of these differences might be questioned. The R${}^{2}$ (0.97) and reproducibility (0.86) of the response is, however, good, and therefore it can be concluded that an effect of the process settings on the diameter does exist. The fact that such small size differences are reproducible underlines the overall reproducibility of the 3D printing process itself.

The significant factors in the effect plot arewere the overlap (0.10 mm), shells (0.09 mm) and layer height (0.06 mm) as can be seen in Figure S3. Significant interactions were the overlap*layer height (0.07 mm), infill*line pattern (−0.06 mm) and shell*overlap (−0.05 mm). The interaction plots are displayed in Figure S4. Overlap had the largest effect on the diameter. As explained previously, the overlap is the degree of infill laid over the innermost shell. Therefore, the printer nozzle deposits material of the infill in the same plane as the previously printed shell, which is, therefore, slightly pushed aside, thus, increasing the diameter. The effect of overlap is more prominent at a high layer height, possibly because more material is deposited at each infill–shell interface, which intensifies the diameter expansion.

The effect of overlap is also more distinct at one shell compared with at three shells. The overlap only refers to the degree of overlay between the infill and the innermost shell, and therefore the volume change will be smaller when additional shells are present, since these hinder any movement of the innermost shell. Lastly, a change from the honey to line pattern will have a larger effect on the diameter at high infill compared with at low infill. This results from the additional hexagons of the honey pattern, which function as additional shells.

#### 3.2.2. Width

For all but one run, the width was lower than 5.5 mm and varied between 5.23 and 5.51 mm over all runs. As such, the differences were again small between different runs (maximally 0.28 mm) with the R${}^{2}$ (0.96) and reproducibility (0.90) of the response being good.

The significant factors were shells (0.10 mm), overlap (0.09 mm), infill (0.08 mm) and layer height (0.05 mm), which are the same main factors and in the same order as for the diameter (except for the infill). No interactions were found (Figure S3). The similarity between both responses can be justified since the width and diameter are properties within the same plane.

#### 3.2.3. Height

The height varied between 4.34 and 4.81 mm over all runs and, as such, the theoretical value of 4.4 mm was often obtained or exceeded. The variation between different runs was also larger (maximally 0.47 mm) compared to the diameter and width response. The reproducibility (0.96) of the response was very good, but the R${}^{2}$ was somewhat lower (0.78).

Moreover, the square test was triggered since the replicates had low values (4.41 and 4.37 mm for the line pattern and 4.38 and 4.34 mm for the honey pattern) in comparison with the other runs (mean height of 4.54 mm) as can be seen in the replicate plot (Figure S5). Therefore, no significant model terms were found. If a predictive model must be constructed for the height response, the current design should be improved in order to identify and estimate the quadratic model term(s).

#### 3.2.4. Weight

A weight range from 90.6 to 223.5 mg can be covered solely by modifying the process settings. This implies that the weight can be more than doubled using the same filament and in silico model. This is in accordance with previous literature, where the weight was almost doubled by changing infill, shell thickness and layer height [37]. The R${}^{2}$ (0.997) and reproducibility (0.99) were excellent for this response.

Significant factors were the infill (66.95 mg), overlap (22.86 mg), shells (10.13 mg), layer height (7.95 mg) and line pattern (−6.50 mg). Significant interactions were the shells*overlap (−13.52 mg), shells*layer height (−12.42 mg), infill*layer height (−9.89 mg), overlap*line pattern (7.87 mg) and infill*shells (−6.88 mg). The effect plot and interaction plot are shown in Figures S3 and S6. The effects of infill and overlap are evident since more material is printed. Many studies have already indicated that infill has a significant influence on the weight [26,32,33,43,54].

As mentioned previously, a shell is a part of the caplet at 100% infill and as such, more material is needed to print more shells. The layer height also increases the weight, since thicker layers consume more material. The line pattern has a negative effect, which means that the weight decreases when the honeycomb is interchanged with the line pattern. Concerning the interactions, the factors overlap, layer height and infill have more effect on the weight at low level of shells. Most likely, the effect of these individual factors is less pronounced when the caplet is already heavier due to a higher number of shells.

#### 3.2.5. Implications for a QbD Approach

In conclusion, a higher level of the factors reduces the deviation from the desired geometry where especially the overlap has the highest impact. Differences between different runs are, however, rather small, and the relevance of these differences might be questioned. In addition, it must be noted that the height of the caplet is a non-linear response.

The ultimate goal of FDM-based 3D-printing is to manufacture flexible dosage forms. One of the pillars of 3D-printing is the possibility to create a caplet with a flexible dose. Therefore, a model was generated for the weight of the tablet containing the infill, shell, overlap, layer height, infill*layer height, shell*overlap and shell*layer height (Figure 7) with an R${}^{2}$ of 0.96, Q${}^{2}$ of 0.85, reproducibility of 0.99 and model validity of 0.52 (Table 2). The effect plot of this model stipulates that the weight is mostly influenced by the infill, followed by overlap and shells.

**Figure 9:**
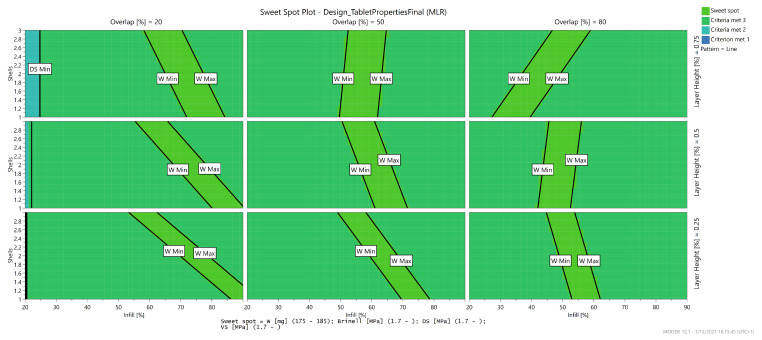
Sweet spot plot presenting the region in the knowledge space where the caplet will meet the user’s specified criteria.

The layer height is only involved in an interaction. This model can be used in combination with the mechanical models to predict the sweet spot of the print settings to produce a caplet with a certain weight and acceptable mechanical properties. A sweet spot plot displays a region within the knowledge space where the responses meet the user’s criteria [49]. An example of a sweet spot plot is given for a caplet with acceptable mechanical properties and a weight between 175 and 185 mg in Figure 9.

### 3.3. Porosity Analysis

An immediate release formulation should rapidly dissolve upon contact with physiological fluids. The first step in disintegration and consequent dissolution is the penetration of liquid in the pores of the caplet. As such, porosity is one of the most important factors facilitating the process [26,55,56]. In FDM 3D-printing, the porosity of the printed object can be very high by selecting a low infill value, e.g., tablets of thermoplastic polyurethanes with 35% porosity or tablets of hydroxypropyl methylcellulose with a porosity of 80% have been produced [26,57,58]. Highly porous products can be produced when a low infill is chosen due to the high viscosity of melts deposited in a layer-by-layer fashion.

Based on the effect plot (Figure S7) for the porosity, the significant factors were the infill (−29.59%), overlap (−11.35%), shells (−5.74%) and layer height (-4.65%). Significant interactions were the overlap*line pattern (−6.74%), shells*overlap (5.70%), infill*shells (4.74%), infill*layer height (4.52%), layer height*line pattern (−4.10%) and shells*layer height (3.98%). Interaction plots are shown in Figure S8. Infill had the most pronounced impact on porosity and was negatively correlated with it. This effect was also noticed in previous research, e.g., the porosity decreased from 67.2% to 39.9%, and the pores were smaller when the infill was increased from 20% to 60% [59].

A similar conclusion was drawn by other researchers [23,26,58]. The overlap also influenced porosity by decreasing the gaps between the infill and shell. The number of shells is negatively correlated with porosity as these are layers with 100% infill, hence, reducing the degree of infill within the tablet [58]. Lastly, layer height is also negatively correlated with the porosity. As less layers are printed at a larger layer height, less interfaces are created between individual layers.

Again, the interactions show that the effects are more pronounced for the line pattern and at low number of shells. This originates from the nature of the honeycomb pattern, as previously mentioned. Additional shells diminish the importance of the other factors by a reduction of the amount of infill within the tablet. Minor interactions include the layer height: when this factor is higher, the effect of infill and shells are less pronounced.

### 3.4. Dissolution Behaviour

The empirical Weibull model was employed to successfully characterize a variety of dissolution profiles and to investigate the time dependency of the release process. The scale parameter α is informative for the time scale of the dissolution process. The shape parameter β, on the other hand, informs about the shape of the dissolution curve, which can be parabolic (β < 1), exponential (β = 1) or sigmoid (β > 1). A more informative parameter is T${}_{d}$, which represents the time interval necessary to dissolve 63.2% of the API present within the caplet [60,61].

The only significant factor in the effect plot (Figure S7) is the infill (115 min), although it must be mentioned that the square test was triggered. Indeed, a higher degree of infill has been associated with a slower release pattern [25,26,43,59,62,63]. The absence of an influence of the number of shells or layer height was also previously reported [26]. On the other hand, other researchers found a significant effect of the number of shells on the dissolution rate [33,37,42]. However, this study investigated delayed release caplets using HPMCAS, HPC and HPMC to mediate release through swelling and diffusion [42].

**Figure 10:**
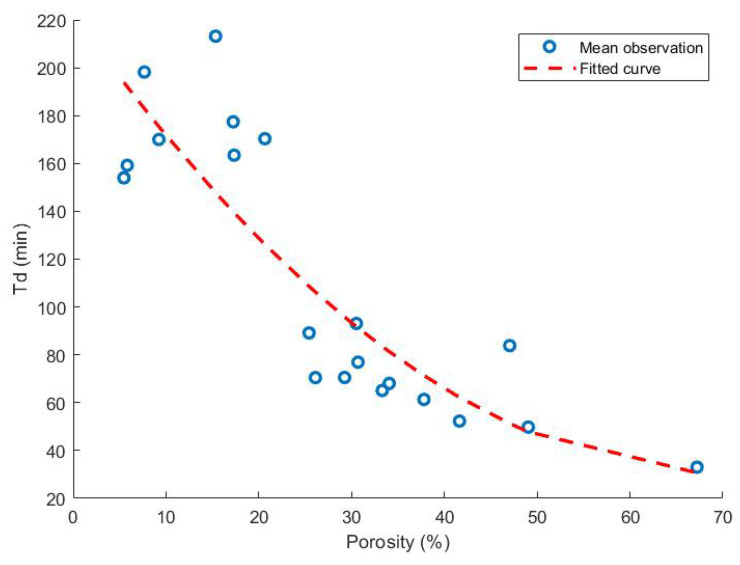
The correlation between the time at which 63.2% of the API is dissolved (Td) and the porosity as measured by helium pycnometry. Reported observations are the mean values of three repetitions of each run. Second order polynomial fitting was performed with an obtained R${}^{2}$ of 0.75.

The correlation between the dissolution parameter T${}_{d}$ and the porosity as measured by He pycnometry is presented in Figure 10 with second order polynomial fitting. Earlier studies have confirmed the correlation between both parameters, e.g., one study noted a decline from 96.9% API released after 45 min to 80.9% when the porosity was decreased from 67.2% to 39.9% [56,59].

From Figure 10, it seems that a larger variation exists especially at low porosity levels. One could argue that the pore structure within a tablet is more complex and can hardly be described by a single parameter, the total porosity [55]. Most likely, a description of the whole pore structure could offer a deeper insight in the link between the porosity and dissolution behaviour of FDM 3D-printed tablets.

For example, research previously showed that the dissolution profile could not easily be predicted based on the tablet porosity as measured by mercury intrusion porosimetry [64]. Moreover, the dissolution time does not rely solely on the porosity of the final caplet. The hydrophilicity or wetting time, for example, should also be taken into account to accurately predict the dissolution time [56].

### 3.5. Summarized Importance of Each Factor

Based on all the investigated responses, the infill appeared to be the most influential factor. This determines the mechanical properties of the caplet, its dimensions, weight, porosity and dissolution behaviour. Surprisingly, the overlap also influenced the dimensions, weight, mechanical properties and porosity to a large extent. The relevance of this factor is often overlooked, and it would be interesting to include this factor in future studies. The number of shells and the layer height have some or limited importance concerning the dimensions of the resulting caplet. The type of layer pattern was often involved in interactions, but its effect is generally limited and can often be anticipated based on the nature of the pattern itself.

### 3.6. Practical Guidelines for Caplet Optimization

FDM 3D-printing is a flexible technique, where CAD models can be freely adapted and modified based on the user’s need. While it is extremely useful to adapt these settings in order to achieve, e.g., the correct dissolution profile, it is also important to keep in mind that certain adaptations also influence other critical quality attributes of the caplets. Based on the QbD analysis presented in this study, practical guidelines and means to adapt certain parameters in order to achieve the desired outcome are stipulated for medical 3D-printing.

#### 3.6.1. Visual Quality of the Dosage Form

**Figure 11:**
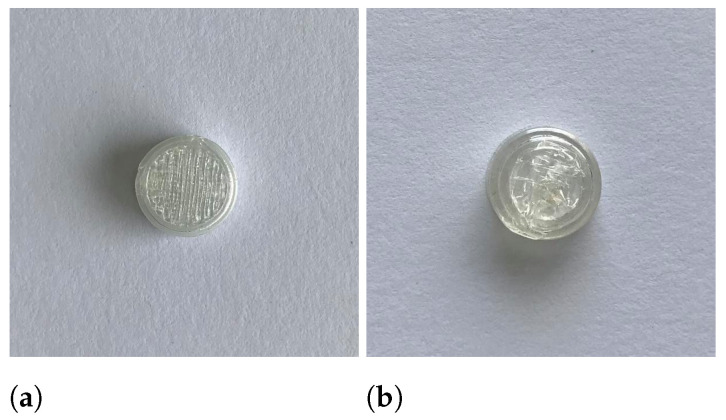
The influence of layer height on the visual quality of the resulting tablet: 0.4 mm (**a**), 0.8 mm (**b**). Reproduced with permission from [18], Elsevier, 2021.

**Figure 12:**
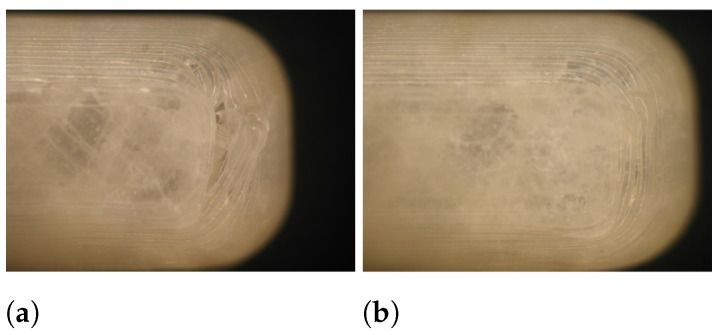
The influence of overlap on the visual quality of the resulting caplet: 10% (**a**), 50% (**b**). Reproduced from [24], MDPI, 2021.

Certain factors influence the visual quality of the resulting dosage form as discussed previously [18,24]. A smaller layer height results in a finer resolution as can be seen in Figure 11, and it is anticipated that a patient would prefer such detail. Moreover, a sufficient overlap is essential to produce qualitative dosage forms with rounded edges (e.g., a caplet) as can be seen in Figure 12. In this specific case, the overlap had to be at least 50% to ensure caplet quality. From the DoE, we learn that overlap and layer height can easily be adapted with limited effect on other quality attributes.

A smaller layer height is detrimental for the mechanical strength of the tablet, but this effect is counteracted if the overlap is increased. Both factors also have a minor influence on the dimensions of the resulting caplet, but it is anticipated that it is not clinically relevant. The weight can vary slightly. The porosity of the caplet will vary although in terms of statistical relevant effects, no influence on the dissolution speed is expected based on the results of this study.

However, it must be noted that this might be of relevance when sustained release dosage forms are developed. In conclusion, overlap and layer height can freely be adapted in order to construct a qualitative dosage form to improve the patient’s acceptance, although special care should be given to dose strength variations.

#### 3.6.2. Acceptable Mechanical Strength

In general, 3D-printed caplets possess acceptable mechanical strength, and this quality attribute poses almost no limits to the sweet spot. However, in order to meet a minimal tensile strength of 1.7 MPa, care should be taken when a low infill (<25%) is combined with a small overlap (<50%) when a layer height of 0.3 mm is used.

#### 3.6.3. Flexible Dosing

FDM 3D-printing is often suggested as a flexible manufacturing tool to produce personalised dosage forms. During this research, we discovered that infill has the largest effect on the weight of caplets when the same external volume is used. However, it must be remembered that infill also largely influences the dissolution behaviour of the caplet. Therefore, small dose adaptations can be done more easily by varying the overlap and number of shells. In order to generate large dose differences, varying the size of the caplet might be a better solution.

#### 3.6.4. Acceptable Release Profile

**Figure 13:**
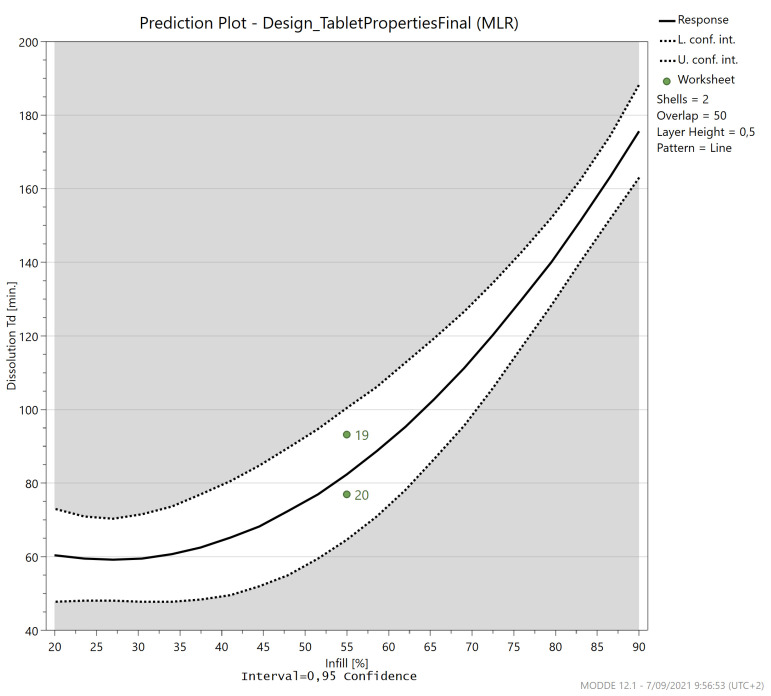
The prediction plot for the dissolution time (Td) in function of the infill (%) when all other factors are kept at their centre value and line pattern is chosen.

The infill is the only significant factor determining the release profile. If all factors are kept at the centre value (0), changing the infill from 20% to 90%, increased the dissolution Td from 60 to 176 min as can be seen in Figure 13. Therefore, the dissolution behaviour can easily be tailored by selecting the appropriate infill percentage. This factor is however connected to all other relevant caplet quality attributes and should be adapted with care.

### 3.7. Limitations of the Current Research

The material properties from the feedstock will also influence the characteristics of the 3D-printed dosage form. For example, it was previously described that the correlation between infill and porosity was material dependent, although the same trend was observed for all investigated materials (ABS, HIPS and PLA) [23]. In another research paper, a linear correlation was found between bonding strength and porosity over a wide range of materials although the exact slope and intercept depended on the intrinsic material properties [50]. Future experiments could help to elucidate this link, although the same trends are expected to prevail over a wide range of material properties.

## 4. Conclusions

This study investigated the effect of various printing parameters (infill, overlap, shells, layer height and infill pattern) on the critical quality attributes of 3D-printed caplets. The factors infill and overlap had the largest influence on most responses, while the number of shells, layer height and layer pattern exerted only a marginal influence. In general, the 3D-printed caplets possessed excellent vertical mechanical strength; however, certain factor combinations should be avoided in order to preserve a minimal diametral strength (1.7 MPa).

The effects of the factors on the dimensions of the caplets are small and are not expected to be clinically relevant. The porosity is a complex response influenced by a number of factors and their interactions but seemed to be correlated with the elastic recovery and dissolution behaviour. The weight and dissolution behaviour were mainly affected by the infill percentage. Therefore, small dose adaptations can easily be made using the overlap and number of shells, while large dose adaptations would preferably be achieved using varying dimensions if the dissolution behaviour must be maintained.

## Figures and Tables

**Table 1:** Overview of factor settings in the experimental design.

Run	Infill (%)	Shells	Overlap (%)	Layer Height	Pattern
1	20	1	20	0.1	Line
2	90	1	20	0.1	Honey
3	20	3	20	0.1	Honey
4	90	3	20	0.1	Line
5	20	1	80	0.1	Honey
6	90	1	80	0.1	Line
7	20	3	80	0.1	Line
8	90	3	80	0.1	Honey
9	20	1	20	0.3	Honey
10	90	1	20	0.3	Line
11	20	3	20	0.3	Line
12	90	3	20	0.3	Honey
13	20	1	80	0.3	Line
14	90	1	80	0.3	Honey
15	20	3	80	0.3	Honey
16	90	3	80	0.3	Line
17	55	2	50	0.2	Honey
18	55	2	50	0.2	Honey
19	55	2	50	0.2	Line
20	55	2	50	0.2	Line

**Table 2:** Overview of the parameters from the models constructed for the Brinell hardness, diametral and vertical strength, weight and dissolution profile.

Effect	R${}^{2}$	Reproducibility	Q${}^{2}$	Validity
Diametral strength	0.94	0.99	0.89	0.39
Vertical strength	0.93	0.96	0.87	0.75
Brinell hardness	0.98	0.95	0.95	0.93
Weight	0.96	0.99	0.85	0.52
Td	0.92	0.95	0.90	0.76

## Data Availability

Not applicable.

## Supplementary Materials

The raw data files, effect plots and interaction plots of each response are available online at https://www.mdpi.com/article/10.3390/pharmaceutics13122068/s1.
