# Supplementary material for: Influence of Print Settings on the Critical Quality Attributes of Extrusion-Based 3D-Printed Caplets: A Quality-by-Design Approach
Source: Pharmaceutics. 2021 Dec 3;13(12):2068. doi: 10.3390/pharmaceutics13122068 (PMC8708825; doi:10.3390/pharmaceutics13122068)
Supplement: Supplementary file 1 [file pharmaceutics-13-02068-s001.zip › pharmaceutics-1457986-sup.pdf]

pharmaceutics

Supplementary Materials: Influence of Print Settings on the Critical Quality Attributes of Extrusion-Based 3D-Printed Caplets: A Quality-by-Design Approach

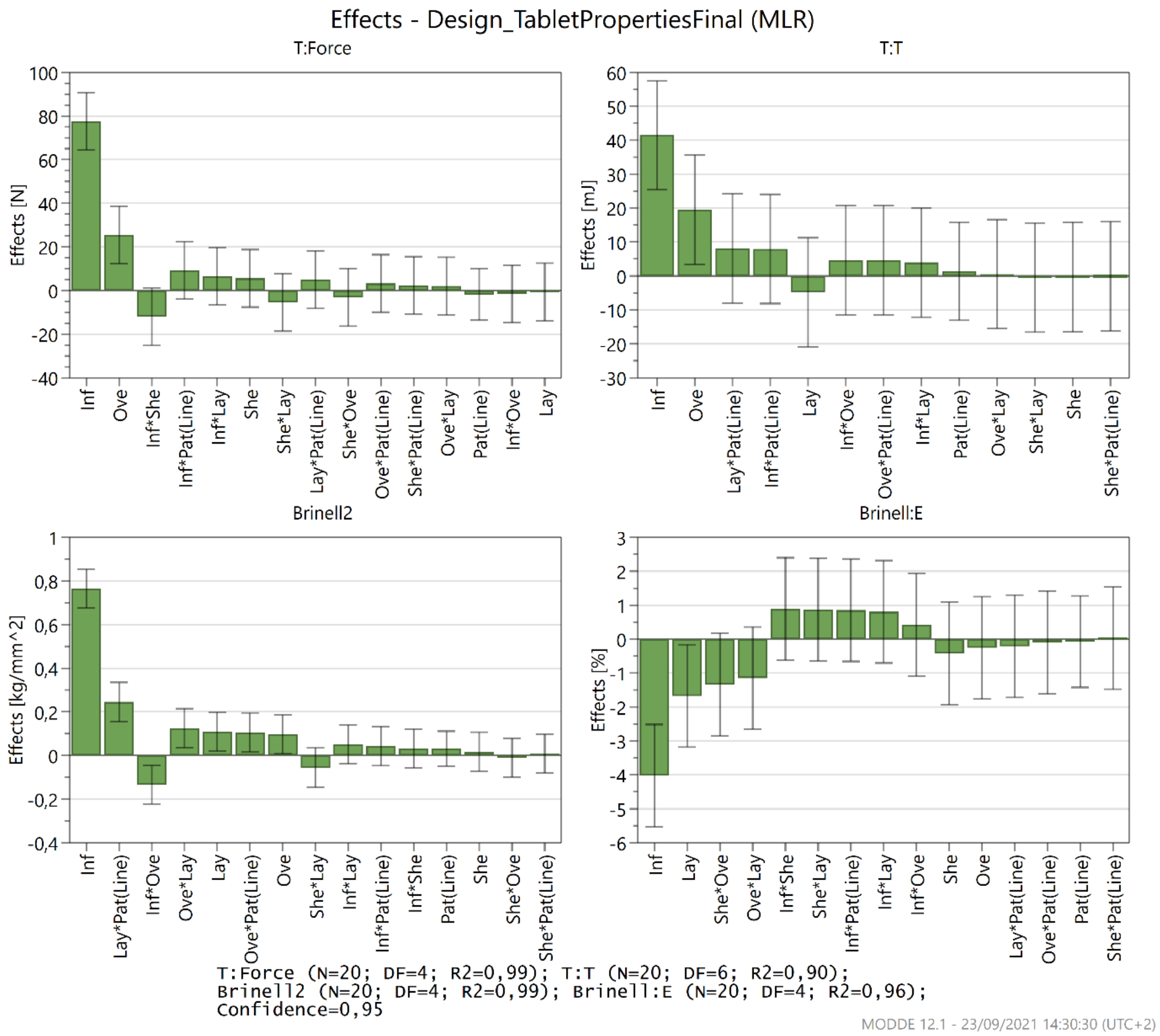

Figure S1. Effect plots of the mechanical properties: peak force of the vertical compression test (T:Force), area under the curve of the vertical compression test (T:T), Brinell hardness (Brinell2) and elasticity as determined during the Brinell hardness test (Brinell:E). The plots include 95% confidence intervals for infill (Inf), overlap (Ove), number of shells (She), layer height (Lay) and infill pattern (Pat) with their interactions (*) displayed as factors.

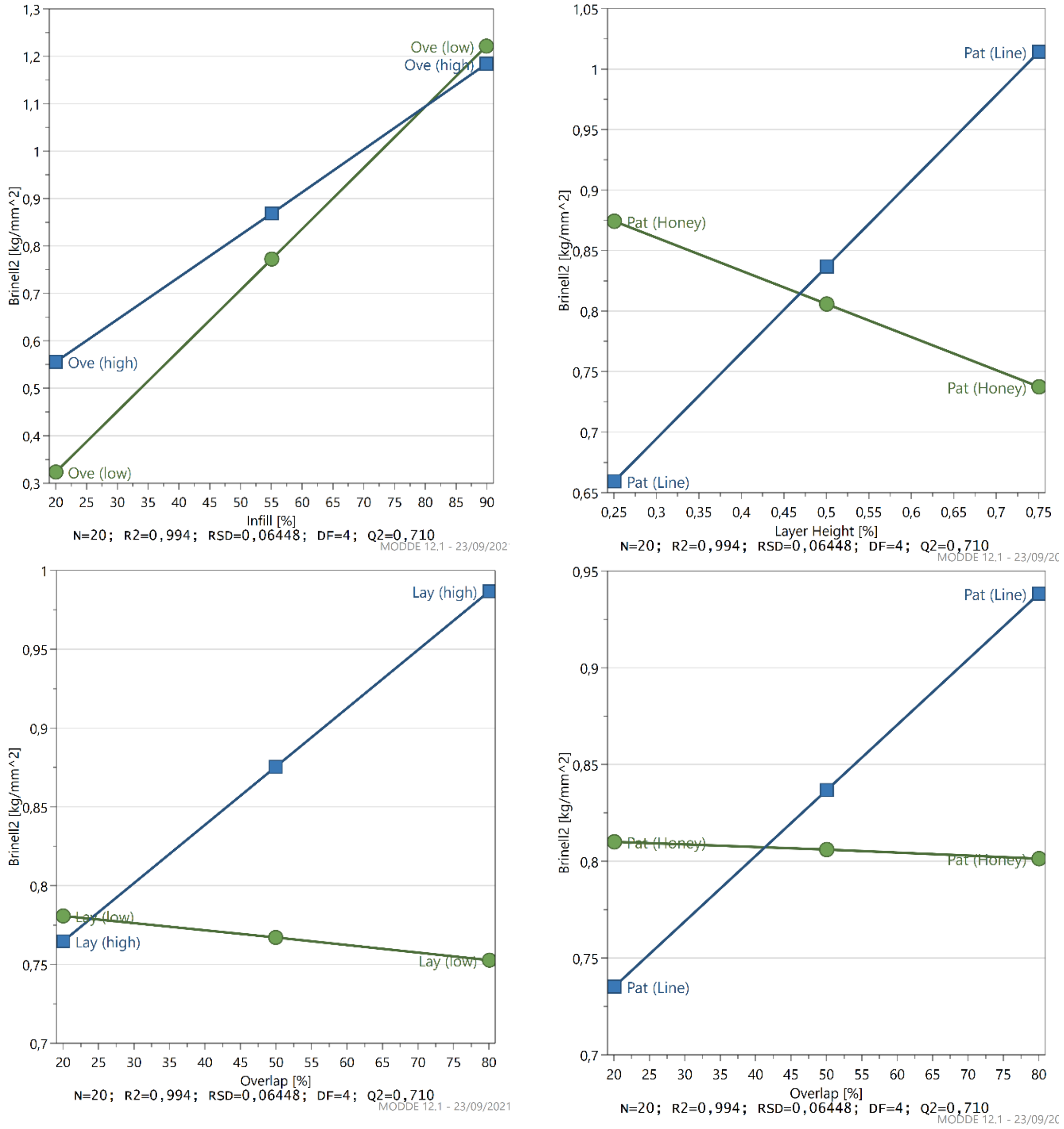

Figure S2. Interaction plots of the Brinell hardness offering a graphical representation of the interaction and its resulting effect. Displayed interactions are the overlap*infill, pattern*layer height, layer height*overlap and pattern*overlap.

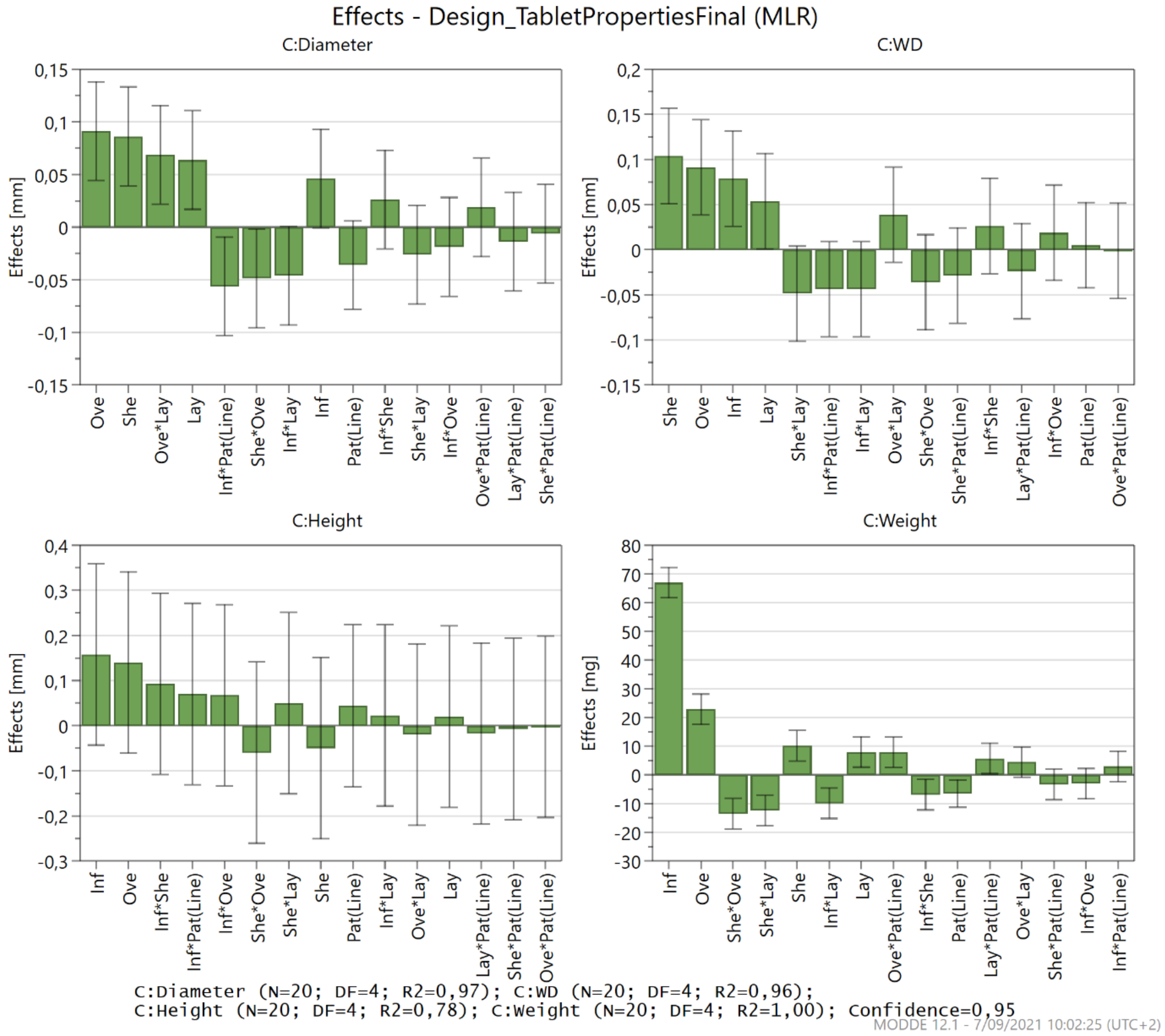

Figure S3. Effect plots for the dimensions: diameter (C:Diameter), width (C:WD), height (C:Height) and weight (C:Weight). The plots include 95% confidence intervals for infill (Inf), overlap (Ove), number of shells (She), layer height (Lay) and infill pattern (Pat) with their interactions (*) displayed as factors.

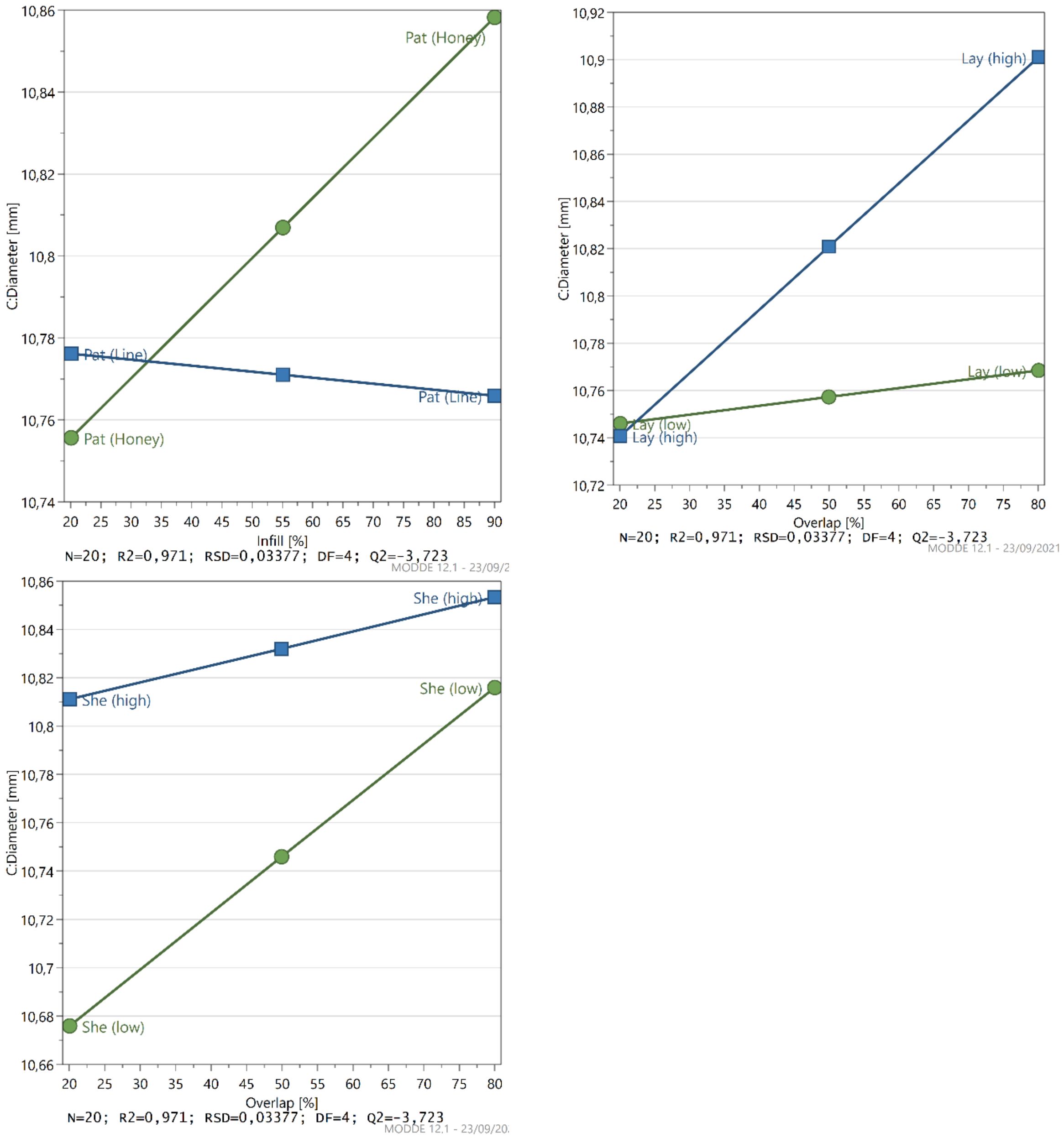

Figure S4. Interaction plots of the diameter offering a graphical representation of the interaction and its resulting effect. Displayed interactions are the pattern*infill, layer height*overlap and shell*overlap.

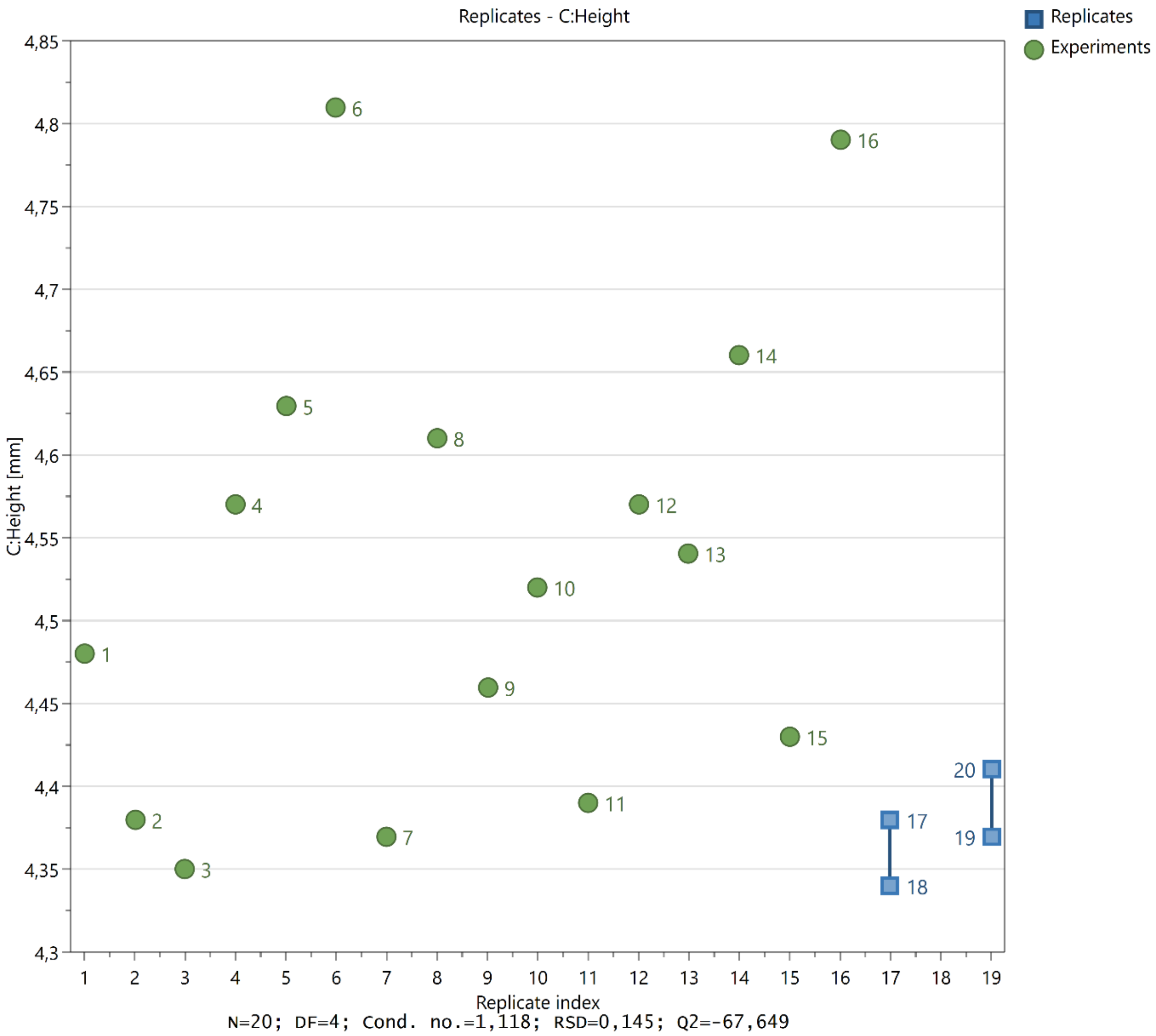

Figure S5. Replicate plot showing the variation for all experiments. Variability of the center points is less than the overall variability, however the position of the center points in the lower part of the plot instead of the middle points out that this response might not be linear.

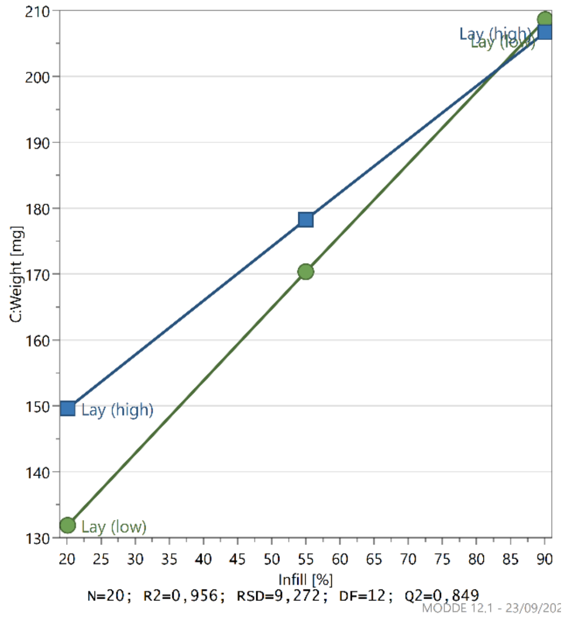

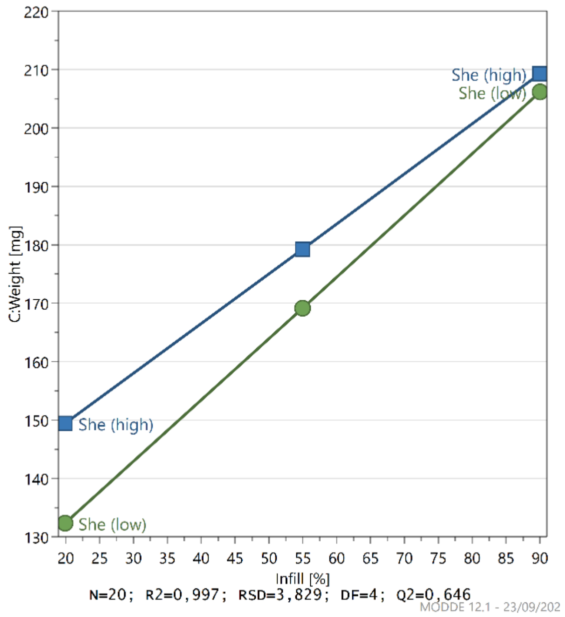

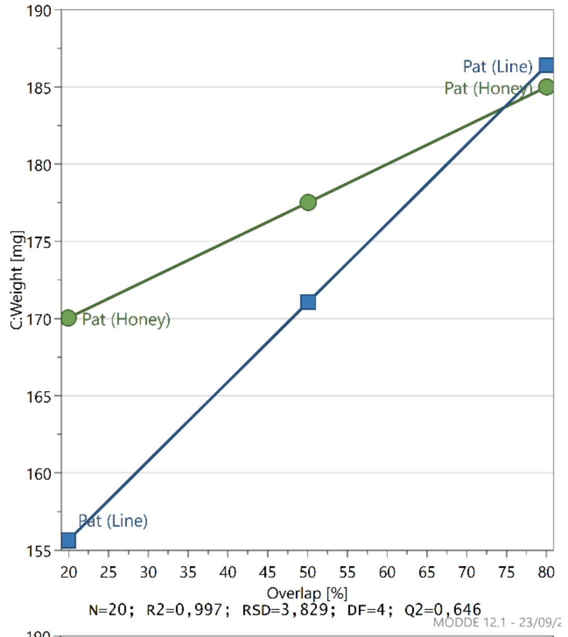

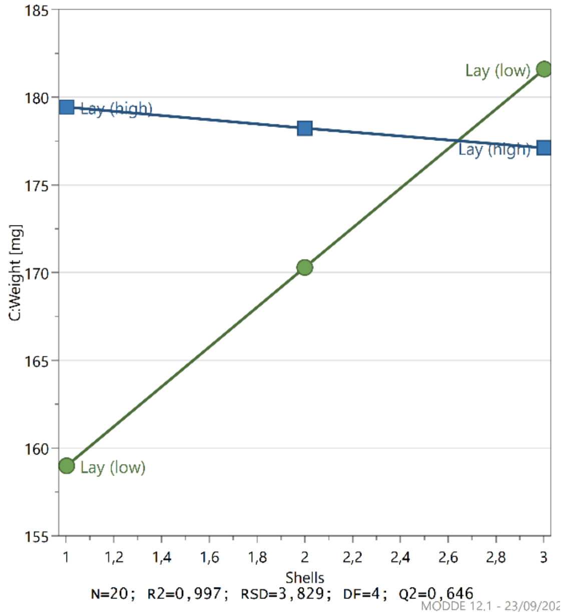

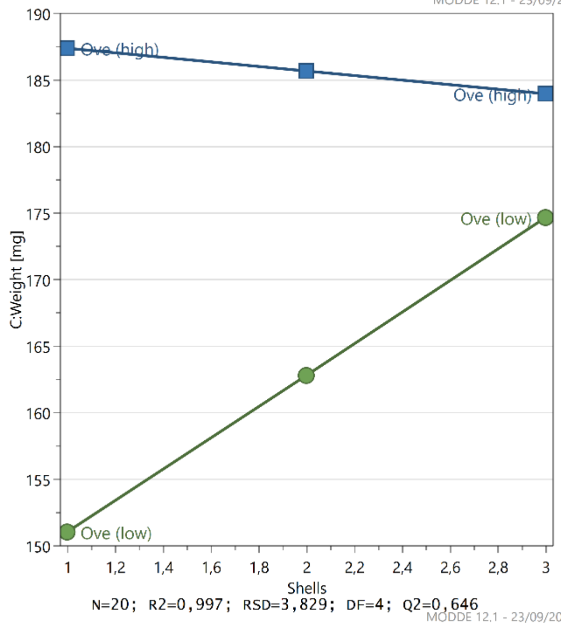

Figure S6. Interaction plots of the weight offering a graphical representation of the interaction and its resulting effect. Displayed interactions are the layer height*infill, shell*infill, pattern*overlap, layer height*shell and overlap*shell.

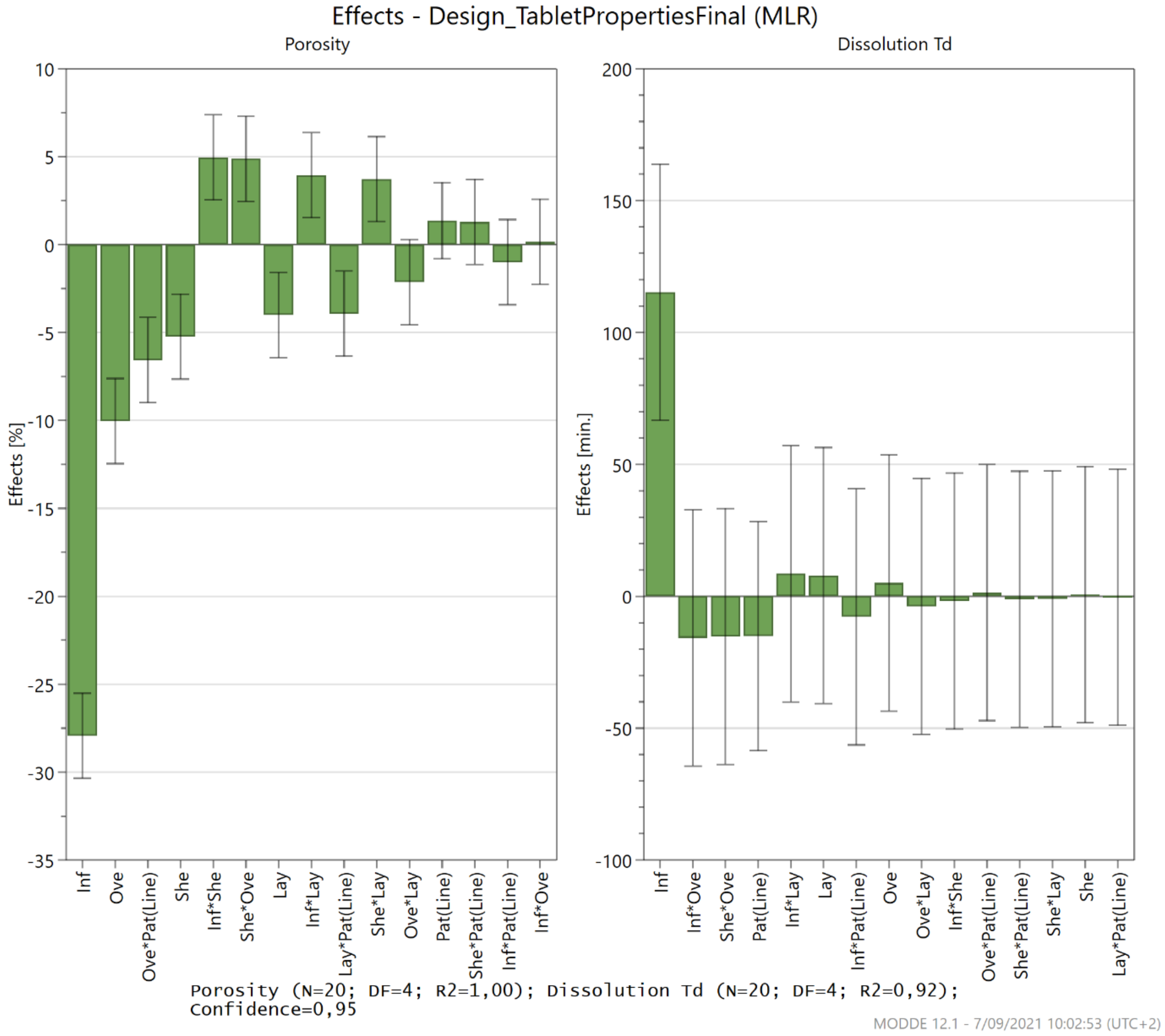

Figure S7. Effect plots of the porosity and dissolution Td parameter. The plots include 95% confidence intervals for infill (Inf), overlap (Ove), number of shells (She), layer height (Lay) and infill pattern (Pat) with their interactions (*) displayed as factors.

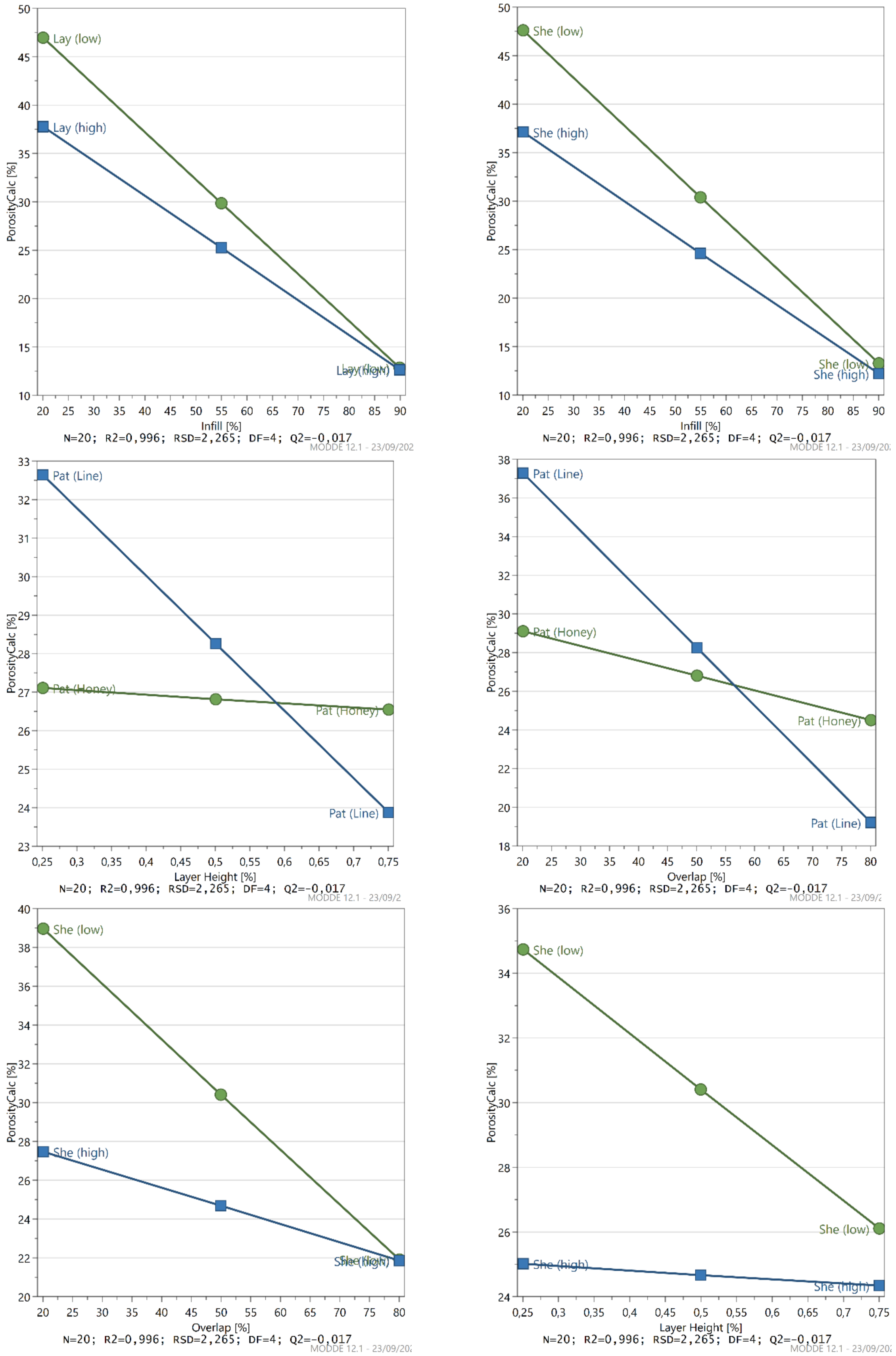

Figure S8. Interaction plots of the porosity offering a graphical representation of the interaction and its resulting effect. Displayed interactions are the layer height*infill, shell*infill, pattern*layer height, pattern*overlap, shell*overlap and shell*layer height.

Table S1. Drug content of the tablets used for dissolution testing. The content was calculated based on the weight of the individual tablets and the experimentally determined content in the hot-melt extruded filament (1.0184 m/m %).

Run	Tablet 1 (mg)	Tablet 2 (mg)	Tablet 3 (mg)
**R1**	9.78	9.82	9.03
**R2**	21.50	20.92	24.25
**R3**	16.43	15.68	15.67
**R4**	20.56	20.70	21.08
**R5**	14.17	13.38	13.36
**R6**	21.44	21.54	21.99
**R7**	15.01	15.22	15.21
**R8**	23.46	23.25	22.94
**R9**	13.02	13.78	13.73
**R10**	20.34	20.85	20.88
**R11**	13.66	13.80	14.15
**R12**	21.30	21.26	21.36
**R13**	17.12	17.56	17.66
**R14**	22.58	23.27	22.93
**R15**	16.08	16.40	16.29
**R16**	22.75	22.95	21.90
**R17**	18.89	18.07	18.21
**R18**	17.03	17.07	17.62
**R19**	17.44	16.95	16.66
**R20**	18.06	17.14	17.67

Table S2. Tabular representation of the significant effect of factors and interactions for the responses under section 3.2: diameter, width, height and weight (n.a. means not applicable).

Factor	Diameter (mm)	Width (mm)	Height (mm)	Weight (mg)
Infill	n.a.	0.08	n.a.	66.95
Shells	0.09	0.10	n.a.	10.13
Overlap	0.10	0.09	n.a.	22.86
Layer height	0.06	0.05	n.a.	7.95
Pattern	n.a.	n.a.	n.a.	-6.50
Overlap*Layer height	0.07	n.a.	n.a.	n.a.
Infill*Line pattern	-0.06	n.a.	n.a.	n.a.
Shell*Overlap	-0.05	n.a.	n.a.	-13.52
Shell*Layer height	n.a.	n.a.	n.a.	-12.42
Infill*Layer height	n.a.	n.a.	n.a.	-9.89
Overlap*Line Pattern	n.a.	n.a.	n.a.	7.87
Shell*Infill	n.a.	n.a.	n.a.	-6.88

Table S3. Raw data (mean) of the investigated caplet properties.
DS = diametral strength, VS = vertical strength, BH = Brinell hardness, BE = Brinell elasticity

**Run**	**Mechanical**				**Dimensional analysis**				**Porosity**	**Dissolution**	**Dose**
	DS MPa	VS MPa	BH MPa	BE %	Diameter mm	Height mm	Width mm	Weight mg	%	Td Min.	mg
1	1.23	0.92	1.50	6.67	10.63	4.48	5.26	90.62	67.22	33.04	9.54
2	6.26	6.69	12.67	0.73	10.76	4.38	5.28	193.29	17.24	177.42	22.22
3	2.68	3.71	5.50	6.86	10.76	4.35	5.35	155.71	34.05	65.10	15.93
4	8.29	8.14	11.14	2.35	10.84	4.57	5.48	206.22	17.34	163.45	20.78
5	1.74	3.86	5.19	9.64	10.69	4.63	5.23	136.57	47.04	83.85	13.64
6	10.10	7.35	9.48	3.94	10.73	4.81	5.40	217.08	5.82	159.21	21.66
7	2.71	4.06	4.08	4.89	10.77	4.37	5.39	146.08	37.80	61.34	15.15
8	9.57	7.58	10.98	1.71	10.89	4.61	5.56	219.77	9.23	170.01	23.22
9	1.35	1.99	2.55	5.73	10.70	4.46	5.29	129.56	49.04	49.79	13.51
10	4.92	7.47	13.22	0.58	10.62	4.52	5.29	192.08	20.65	170.35	20.69
11	1.65	2.54	3.30	5.20	10.76	4.39	5.38	136.94	41.63	52.30	13.87
12	4.67	7.89	11.11	3.00	10.89	4.57	5.47	201.28	15.35	213.19	21.31
13	2.18	3.99	8.44	3.67	10.93	4.54	5.49	173.89	25.42	70.47	17.45
14	8.06	9.59	11.07	0.81	10.92	4.66	5.51	223.51	7.67	198.21	22.93
15	2.47	3.86	4.25	3.54	10.90	4.43	5.46	159.95	33.31	68.04	16.26
16	7.19	8.26	15.14	0.87	10.86	4.79	5.49	211.71	5.46	153.98	22.53
17	4.79	6.50	8.51	2.71	10.81	4.38	5.38	181.84	26.10	89.15	18.39
18	4.42	5.81	7.23	2.89	10.75	4.34	5.33	173.78	29.24	70.53	17.24
19	3.04	5.16	7.38	3.56	10.80	4.37	5.38	168.28	30.51	93.11	17.02
20	3.39	5.87	8.42	5.16	10.77	4.41	5.35	167.38	30.69	76.93	17.62
